# Multivariate Modelling and Prediction of High-Frequency Sensor-Based Cerebral Physiologic Signals: Narrative Review of Machine Learning Methodologies

**DOI:** 10.3390/s24248148

**Published:** 2024-12-20

**Authors:** Nuray Vakitbilir, Abrar Islam, Alwyn Gomez, Kevin Y. Stein, Logan Froese, Tobias Bergmann, Amanjyot Singh Sainbhi, Davis McClarty, Rahul Raj, Frederick A. Zeiler

**Affiliations:** 1 Department of Biomedical Engineering, Price Faculty of Engineering, University of Manitoba, Winnipeg, MB R3T 5V6, Canada; islama9@myumanitoba.ca (A.I.); steink34@myumanitoba.ca (K.Y.S.); amanjyot.s.sainbhi@gmail.com (A.S.S.); frederick.zeiler@umanitoba.ca (F.A.Z.); 2 Section of Neurosurgery, Department of Surgery, Rady Faculty of Health Sciences, University of Manitoba, Winnipeg, MB R3A 1R9, Canada; 3 Department of Human Anatomy and Cell Science, Rady Faculty of Health Sciences, University of Manitoba, Winnipeg, MB R3E 0J9, Canada; 4 Department of Clinical Neuroscience, Karolinska Institutet, 171 77 Stockholm, Sweden; log.froese@gmail.com; 5 Undergraduate Engineering, Price Faculty of Engineering, University of Manitoba, Winnipeg, MB R3T 5V6, Canada; bergmant@myumanitoba.ca; 6 Undergraduate Medicine, College of Medicine, Rady Faculty of Health Sciences, Winnipeg, MB R3E 3P5, Canada; mcclartd@myumanitoba.ca; 7 Department of Neurosurgery, University of Helsinki, 00100 Helsinki, Finland; rahul.raj@hus.fi; 8 Pan Am Clinic Foundation, Winnipeg, MB R3M 3E4, Canada

**Keywords:** cerebral physiologic signals, computational models of cerebral physiology, high-dimensional cerebral data analysis, multivariate machine learning models

## Abstract

Monitoring cerebral oxygenation and metabolism, using a combination of invasive and non-invasive sensors, is vital due to frequent disruptions in hemodynamic regulation across various diseases. These sensors generate continuous high-frequency data streams, including intracranial pressure (ICP) and cerebral perfusion pressure (CPP), providing real-time insights into cerebral function. Analyzing these signals is crucial for understanding complex brain processes, identifying subtle patterns, and detecting anomalies. Computational models play an essential role in linking sensor-derived signals to the underlying physiological state of the brain. Multivariate machine learning models have proven particularly effective in this domain, capturing intricate relationships among multiple variables simultaneously and enabling the accurate modeling of cerebral physiologic signals. These models facilitate the development of advanced diagnostic and prognostic tools, promote patient-specific interventions, and improve therapeutic outcomes. Additionally, machine learning models offer great flexibility, allowing different models to be combined synergistically to address complex challenges in sensor-based data analysis. Ensemble learning techniques, which aggregate predictions from diverse models, further enhance predictive accuracy and robustness. This review explores the use of multivariate machine learning models in cerebral physiology as a whole, with an emphasis on sensor-derived signals related to hemodynamics, cerebral oxygenation, metabolism, and other modalities such as electroencephalography (EEG) and functional near-infrared spectroscopy (fNIRS) where applicable. It will detail the operational principles, mathematical foundations, and clinical implications of these models, providing a deeper understanding of their significance in monitoring cerebral function.

## 1. Introduction

The high-frequency measurements of cerebral physiologic parameters provide real-time insights crucial for the precise assessments of cerebral function, enabling the capture of rapid fluctuations, the detection of subtle changes, and the guidance of real-time interventions, making them indispensable for understanding, monitoring, and treating cerebral disorders [1,2,3]. These high-frequency signals are generated by a combination of invasive and non-invasive sensor technologies, which monitor cerebral oxygenation, metabolism, and hemodynamic parameters, including intracranial pressure (ICP) and cerebral perfusion pressure (CPP) [1,4,5]. Additionally, neural activity signals recorded by sensor-based modalities such as electroencephalography (EEG), functional near-infrared spectroscopy (fNIRS), and magnetoencephalography (MEG) provide complementary data that enrich the understanding of cerebral function, particularly when integrated with multivariate analysis approaches. Understanding and analyzing these signals are pivotal for grasping the complexities of cerebral processes, as it enables the identification of intricate patterns and accurately pinpoint anomalies [6]. The development of computational models of cerebral physiology plays a vital role in exploring the connections between measurable signals and the underlying physiological state. However, the complexity and multidimensionality of cerebral data poses significant challenges for traditional analytical methods [2,6]. Herein lies the importance of leveraging multivariate machine learning approaches to unlock the full potential of these signals.

Multivariate models, particularly machine learning models, have emerged as indispensable tools for cerebral physiological signal analysis due to their capacity to capture intricate relationships among multiple variables recorded by different sensors simultaneously. Unlike conventional univariate methods, which analyze individual signals in isolation and may overlook subtle yet clinically relevant patterns, multivariate models excel in integrating diverse physiological parameters together, extracting meaningful information from complex datasets [7]. This capability is particularly valuable in neuroimaging studies, where numerous factors, measured by different sensors, contribute to brain activity and dynamics [8]. Moreover, multivariate machine learning facilitates accurate modeling of cerebral physiologic signals, effectively capturing complex patterns in cerebral data, delineate functional brain networks, and can help identify biomarkers of neurological disorders [9,10]. Such modeling approaches not only enhance our understanding of brain physiology but also hold promise for developing novel diagnostic and prognostic tools for a wide range of neurological conditions.

Furthermore, the predictive capabilities of multivariate machine learning are instrumental in advancing personalized medicine and treatment optimization in neurology [1,5]. By harnessing large-scale datasets encompassing diverse patient populations and clinical variables, these models can forecast disease progression, treatment response, and patient outcomes with unprecedented accuracy [11,12,13]. In the context of cerebral physiologic signals, predictive modeling enables patient-specific intervention, thereby optimizing therapeutic efficacy and improving patient care [14].

Additionally, machine learning models exhibit remarkable versatility, particularly in their capacity to be combined synergistically to tackle complex challenges in cerebral physiological signal analysis and modeling. By integrating multiple machine learning algorithms, the strengths of each model can be harnessed to address specific aspects of cerebral data analysis comprehensively [15,16]. For instance, ensemble learning techniques, such as random forests or gradient boosting, enable the aggregation of predictions from diverse base learners, enhancing predictive accuracy and robustness [17]. In the context of cerebral physiologic signals, combining various machine learning models allows for a more nuanced understanding of brain function and pathology by leveraging the complementary strengths of different approaches, as well as allowing for the mitigation of the limitations of individual models, such as overfitting or bias, resulting in more reliable and interpretable results [18].

**Figure 1:**
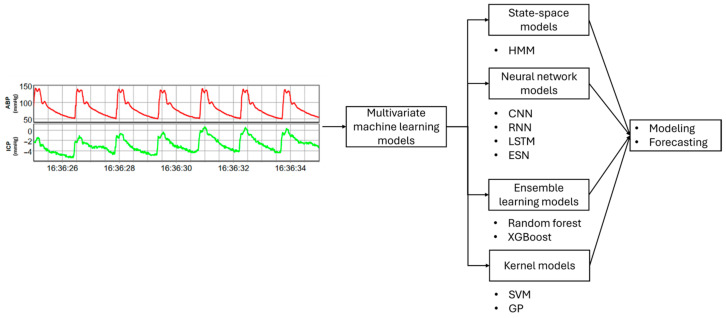
An overview illustrating the pathway from raw data to the final model through various machine learning models.

In this study, hidden Markov models (HMM), convolutional neural networks (CNNs), long short-term memory (LSTM) networks, recurrent neural networks (RNNs), echo state networks (ESNs), random forests, XGBoost, support vector machines (SVMs), and Gaussian processes (GPs) are studied. Among these, CNNs, LSTMs, and RNNs fall under the category of deep learning, a subfield of machine learning. These models not only serve as powerful tools for automated feature extraction but are also widely employed as standalone predictive models, learning hierarchical representations directly from raw data. Their inclusion in this study highlights their versatility and importance within the broader machine learning modeling. Figure 1 offers a brief summary of the process from raw data to modeling with various machine learning models, highlighting the key steps involved. The models included in this study were chosen based on their ability to handle the complexity, sequential nature, and non-linear dynamics of the dataset. Classical models such as k-nearest neighbors (k-NNs), logistic regression, linear regression, and naïve Bayes were excluded due to their limitations in modeling temporal dependencies, non-linear relationships, and high-dimensional data effectively. Additionally, the chosen models align with state-of-the-art approaches for similar applications, ensuring robust and interpretable results.

This review aims to provide a thorough investigation of commonly used multivariate machine learning models, categorized as state-space models, neural networks, ensemble learning methods, and kernel methods, which are also summarized in Table 1. By delving into their operational principles and mathematical formulations, this narrative seeks to clarify the complexities associated with these modeling approaches. Moreover, the discussion will not only cover theoretical foundations but also explore practical applications and clinical implications of these models. We conducted a literature search to summarize research from the past 10 years, focusing specifically on the multivariate applications of machine learning models to cerebral physiological signals. To gather the relevant literature, we used a targeted search strategy in PubMed, employing keywords related to machine learning models and cerebral physiological signals. The selected studies were analyzed by categorizing them based on the models used, the signals analyzed, and the purpose of using the model. These studies were then included to provide insight into the current use of machine learning models in this area of research, highlighting how these models are being applied in practice. By bridging theoretical concepts and practical implementations, this review examines how modern machine learning methodologies can be utilized to model cerebral physiologic signals, offering insights into their potential applications in analyzing and interpreting sensor-derived signals.

## 2. Methods

### 2.1. Multivariate State-Space Models

Multivariate state space models are a class of statistical models used to represent complex systems characterized by multiple interacting variables evolving over time which are particularly useful for analyzing sequential data where observations are influenced by latent or unobservable states [19]. In a multivariate state space model, the observed data are assumed to be generated by a process that evolves through a series of hidden states over time, and each hidden state is associated with a multivariate observation, capturing the relationships between different variables [20]. Multivariate state space models offer a flexible framework for modeling temporal dependencies, capturing non-linear relationships, and handling missing or noisy data.

#### Hidden Markov Model (HMM)

The HMM is a versatile statistical modeling approach applicable to ‘linear’ challenges, such as sequences or time series, which assumes the existence of an underlying, unobservable (hidden) state sequence influencing the observed data [21]. The HMM is characterized by two main components: the observable states, which are the directly measurable or observable aspects of the system being modeled, and the hidden states, which represent unobserved variables that capture underlying structures or dynamics in the data sequence. HMM assigns transition probabilities to states, with each state emitting symbols based on its corresponding emission probability [22]. The probability of transitioning from one hidden state to another is described by the state transition probabilities, which is denoted by Equation (1), where *A* represents a transition matrix, *a_ij_* represents the probability of transitioning from hidden state *i* at time *t* to hidden state *j* at time *t*+1, and *S* represent a set of hidden states [22].

(1) $$A=a_{ij}=\left\{\left.P\left(\left.X_{t+1}=S_{j}\right|X_{t}=S_{i}\right)\right|1\leq i,j\leq n\right\},$$

The emission probabilities describe the probability of observing a particular observable state given the current hidden state. These probabilities are modeled using an emission matrix *B* as shown in Equation (2), where *O*(*t*) represents m-observable symbols and *b_j_*(*t*) represents the probability of emitting symbol *O*(*t*) from state *j*.

(2) $$B=b_{j}\left(t\right)=\{\left.P\left(\left.O\left(t\right)\right|X\left(t\right)=S_{j}\right)\right|1\leq j\leq n\},$$

The initial state distribution describes the probability of starting in each hidden state. This distribution is modeled using an initial state vector *π*, denoted by Equation (3), where *π_i_* represents the probability of starting in hidden state *i*.

(3) $$\pi =\{\pi_{i}=P\left(X_{l}=S_{i}\right)|1\leq i\leq n\},$$

With these components, the probability of observing a sequence of observable states can be computed by *O*(*t*) = (*o*_1_,*o*_2_,...,*o*_m_).

HMMs offer flexible modeling of diverse sequential data types and can capture temporal dependencies and transitions effectively [22]. Moreover, HMMs provide a probabilistic framework for robust inference and uncertainty estimation, making them valuable in various domains such as speech recognition, natural language processing, and signal processing. HMMs also offer interpretability by revealing insights into hidden state dynamics and feature extraction capabilities for capturing relevant patterns [22]. However, they assume a Markovian property that may not hold for complex systems and require specifying the number of hidden states beforehand, which can be challenging when the true number of states is unknown [23]. Additionally, HMMs may struggle to capture long-term dependencies in data and face computational challenges with high-dimensional data [22]. Sensitivity to initialization and parameter tuning, as well as vulnerability to overfitting, are also potential drawbacks [24]. Despite these limitations, HMMs remain a valuable tool for modeling sequential data and understanding dynamic processes in various fields. HMMs are increasingly being utilized for the analysis of cerebral physiologic signals, as they provide a robust framework for modeling the temporal dynamics and underlying states that generate these complex signals [25,26,27].

### 2.2. Neural Networks

**Figure 2:**
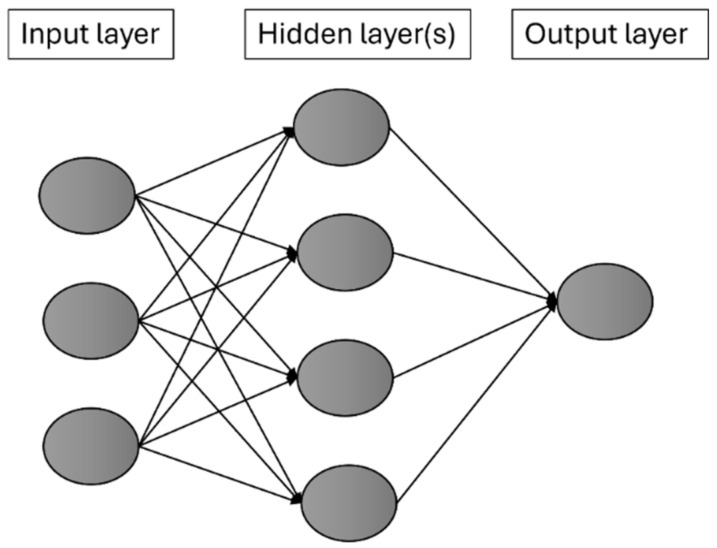
A general neural network architecture.

Neural networks are a class of machine learning models inspired by the structure and function of the human brain. Comprising interconnected nodes, or neurons, organized into layers, neural networks are adept at learning complex patterns and relationships within data. Input data are fed into the network, processed through a series of hidden layers, and transformed into output predictions. Each neuron applies a mathematical operation to its inputs, with parameters (weights and biases) that are learned during training to minimize the difference between predicted and actual outcomes [28]. They come in various architectures, including feedforward, convolutional, recurrent, and attention-based networks, each tailored to specific types of data and tasks. Despite their flexibility, scalability, and ability to learn complex representations, neural networks can be computationally intensive and require large amounts of labeled data for effective training [29]. Figure 2 represents a simplified general structure for neural network architecture. Note that the output layer may include more nodes depending on the task, such as regression or classification. Additionally, the hidden layer might consist of more than one layer.

#### 2.2.1. Convolutional Neural Network (CNN)

CNNs are a type of neural network architecture that are particularly effective at capturing spatial hierarchies of features within data, commonly images, through the use of convolutional layers, pooling layers, and fully connected layers [30]. In a CNN, the convolutional layer applies a series of filters, also known as kernels, to the input data where each filter detects specific features, such as edges or textures, by performing element-wise multiplications and summations across local regions to compute different feature maps [31]. The input is convolved using a learned kernel and subsequently applying a non-linear activation function to the convolved outcomes, resulting in the creation of a new feature map [32]. Multiple feature maps are then obtained through the utilization of various kernels. The feature value can be mathematically computed, as illustrated by Equation (4), where $w_{k}^{l}$ is the weight vector applied to the input and $b_{k}^{l}$ is the bias term of the *k*-th filter of the *l*-th layer, $x_{i,j}^{l}$ is the input and $z_{i,j}^{l}$ represents the feature map at location (*i,j*) of the *l*-th layer [32].

(4) $$z_{i,j,k}^{l}={w_{k}^{l}}^{T}x_{i,j}^{l}+b_{k}^{l},$$

After convolution, the output is typically passed through a non-linear activation function such as sigmoid, hyperbolic tangent function (tanh) or rectified linear unit (ReLU), as presented as given in Equation (5), where $a_{i,j,k}^{l}$ represents the activation value.

(5) $$a_{i,j,k}^{l}=a(z_{i,j,k}^{l}),$$

The pooling layer, typically situated between two convolutional layers, aims to attain shift invariance through the reduction in feature map resolution. Each feature map within the pooling layer is linked to its corresponding feature map from the preceding convolutional layer. Common pooling operations include max pooling and average pooling. A general pooling layer is presented in Equation (6).

(6) $$y_{i,j,k}^{l}=pool(a_{i,j,k}^{l}),$$

Finally, the output from the convolutional and pooling layers is flattened and passed through one or more fully connected layers, which perform classification or regression tasks. These layers, exampled in Equation (7), where *A_f_* represents the flattened output from the previous layers, use weights, *w*, and biases, *b*, to compute the final output *o*, typically followed by a softmax activation function for classification tasks:

(7) $$o=softmax(w*A_{f}+b),$$

The softmax activation function converts raw scores into a probability distribution over multiple classes for multi-class classification tasks. It provides interpretable outputs, and allows for efficient training through gradient-based optimization through Equation (8), where *x* is the vector of the output of previous layer, *K* represents the vector length, and *j* and *i* are indices of a vector element and corresponding softmax output element, respectively [33].

(8) $${f(x)}_{i}=\frac{e^{x_{i}}}{\sum_{j=1}^{K}e^{x_{j}}},$$

CNN models leverage convolutional layers to extract hierarchical features generally from input images, followed by pooling layers for dimensionality reduction and fully connected layers for classification or regression. During the training process, various optimization methods, including the optimization methods such as stochastic gradient descent (SGD), Adam, AdaDelta, Adabelief, and AdaMax, can be employed to adjust the parameters of the network, including the weights and biases, in order to minimize the difference between the predicted outputs and the ground truth labels [34]. Each optimization method has its own advantages and disadvantages, and the choice of method depends on factors such as the specific problem, the size and complexity of the dataset, and computational resources available. Experimentation and tuning are often required to determine the most effective optimization method for a given task.

CNN models have the ability to automatically learn hierarchical representations of data, achieve translation-invariant feature detection, and efficiently process high-dimensional data like images and videos; however, they may struggle with understanding spatial context in certain cases, and they can be challenging to apply to irregular data structures [35]. Additionally, CNNs benefit from parameter sharing and sparse connectivity, enabling more efficient learning and inferences [36]. They are also parallelizable, facilitating fast computation and training on large-scale datasets, and can leverage transfer learning to accelerate model training [30]. On the other hand, they may lack interpretability of learned features, require large amounts of labeled data for training, and be sensitive to variations in hyperparameters [31]. Additionally, designing complex CNN architectures and addressing computational requirements for training and inference can also pose challenges as a balance between model complexity, computational resources, training time, generalization performance, and deployment constraints is required [35].

#### 2.2.2. Recurrent Neural Network (RNN)

RNNs are a type of neural network architecture specifically designed for sequential data processing. RNNs have connections that form directed cycles, allowing them to maintain an internal memory or hidden state that captures information about previous inputs [37]. The hidden state *h_t_* at time step *t* in an RNN can be computed as presented in Equation (9), where *f* is the activation function, such as sigmoid, tanh, or ReLU; *W_hh_* is the weight matrix for the recurrent connections; *h*_*t*−1_ is the hidden state from the previous time step; *x_t_* is the input at time step *t*; *W_xh_* is the weight matrix for connections from *x_t_*; and *b_h_* is the bias vector [38].

(9) $$h_{t}=f(W_{hh}*h_{t-1}+W_{xh}*x_{t}+b_{h}),$$

The output of the RNN at each time step *t* can then be computed as given in Equation (10), where *y_t_* is the output at time step *t*, *W_hy_* is the weight matrix for the connections from the hidden state to the output, and *b_y_* is the bias vector.

(10) $$y_{t}=W_{hy}*h_{t}+b_{h},$$

During training, the parameters (weights and biases) of RNNs are learned using backpropagation through time [38]. RNNs can model temporal dynamics, process variable-length inputs, and share parameters for efficient learning of long-term dependencies [38]. They naturally represent sequential data and maintain stateful memory, enabling them to capture context across time steps and support gradient propagation through backpropagation; however, they suffer from vanishing or exploding gradients during training, limiting their ability to capture long-term dependencies effectively [37]. Short-term memory limitations can impact their performance on tasks requiring an understanding of long-range contexts, and sequential computation slows training and inference, hindering scalability [29]. RNNs are also sensitive to hyperparameters and can exhibit training instability with large datasets or complex architectures, limiting their effectiveness in certain scenarios [37].

#### 2.2.3. Long Short-Term Memory (LSTM)

LSTM networks are a specialized type of RNN architecture designed to address the vanishing gradient problem and capture long-term dependencies in sequential data [16]. LSTMs introduce memory cells with gated units, which allows them to selectively retain or forget information over time enabling LSTMs to learn and remember important information over long sequences, making them well-suited for tasks such as time series prediction [39]. The key components of an LSTM cell include the input gate *i_t_*, forget gate *f_t_*, output gate *o_t_*, cell state *c_t_*, intermediate state *g_t_*, and hidden state *h_t_*, which are updated at each time step *t*. The equations governing the computation of these components are given from Equations (11)–(16), where *x_t_* is the input at time step *t*; *h_t_*_−1_ is the hidden state from the previous time step; *W* and *b* are the weights and biases for the input gate (*i*), output gate (*o*), cell state (*c*), and intermediate state (*g*); *σ* is the sigmoid activation function; and ⊙ represents element-wise multiplication [40].

(11) $$i_{t}=\sigma \left(W_{xi}x_{t}+W_{hi}h_{t-1}+W_{ci}c_{t-1}+b_{i}\right),$$

(12) $$f_{t}=\sigma \left(W_{xf}x_{t}+W_{hf}h_{t-1}+W_{cf}c_{t-1}+b_{f}\right),$$

(13) $$o_{t}=\sigma \left(W_{xo}x_{t}+W_{ho}h_{t-1}+W_{co}c_{t-1}+b_{o}\right),$$

(14) $$g_{t}=tanh\left(W_{xg}x_{t}+W_{hg}h_{t-1}+b_{g}\right),$$

(15) $$c_{t}=f_{t}⨀c_{t-1}+i_{t}⨀{\tilde{c}}_{t}),$$

(16) $$h_{t}=o_{t}⨀tanh(c_{t}),$$

LSTMs excel in learning and retaining information over long sequences, making them ideal for tasks involving temporal context, and they address the vanishing gradient problem of traditional RNNs, ensuring stable training [40]. LSTMs utilize gating mechanisms to regulate information flow and maintain an internal memory cell for context retention [41]. While versatile across various sequential tasks, LSTMs are complex, requiring careful hyperparameter tuning, and may suffer from overfitting. Interpreting LSTM mechanisms can be challenging, hindering performance diagnosis [42]. Despite their proficiency, LSTMs may struggle with very long sequences due to memory limitations and can still face gradient explosion issues during training, especially with deep architectures [41].

#### 2.2.4. Echo State Network (ESN)

ESNs are a type of RNN renowned for their simplicity and effectiveness in handling temporal data [43]. The defining feature of ESNs is their “reservoir”—a fixed, random, and sparse recurrent network that transforms the input signals into high-dimensional dynamic states [44]. The reservoir is made of large recurrently connected neurons, often arranged in a random or fixed structure. The defining characteristic of ESNs lies in their training method, where only the output weights are learned while the internal connections remain fixed [43]. This simplifies the training process and prevents issues such as vanishing or exploding gradients. Typical update equations for ESN are given in Equations (17) and (18), where *x*(*t*) represents the state of the reservoir at time *t*; *y(t)* represents the output value; *W_in_*, *W_res_* and *W_out_* are the input, recurrent, and readout weights, respectively; *u(t)* represents the input value at time *t*; and *f* is a non-linear activation function, such as sigmoid or tan [45,46].

(17) $$x(t)=f\left(W_{in}u\left(t\right)+W_{res}x\left(t-1\right)\right),$$

(18) $$y\left(t\right)=W_{out}x(t),$$

The effectiveness of ESN lies in its ability to generate complex temporal dynamics from simple input–output mappings, making it suitable for tasks such as time-series prediction and signal processing [46]. ESNs offer a computationally efficient alternative to traditional RNNs and LSTMs for tasks involving temporal data, leveraging a fixed reservoir to generate dynamic representations while simplifying the training process [47]. However, the performance of ESNs can be highly sensitive to the design of the reservoir, including its size, connectivity, and spectral radius, making the optimization of these parameters challenging for specific tasks [48]. Since the reservoir is fixed and untrained, it may not always align well with the particular characteristics of the input data, limiting adaptability [49]. Large reservoirs can consume significant memory resources, posing a challenge for memory-intensive applications. Additionally, the fixed reservoir cannot adapt to new data patterns during training, which might limit the network’s effectiveness in dynamic environments [46]. ESNs may struggle with very large-scale problems or tasks requiring highly precise temporal modeling, where the advantages of traditional RNNs or LSTMs might become more pronounced.

### 2.3. Ensemble Learning Models

**Figure 3:**
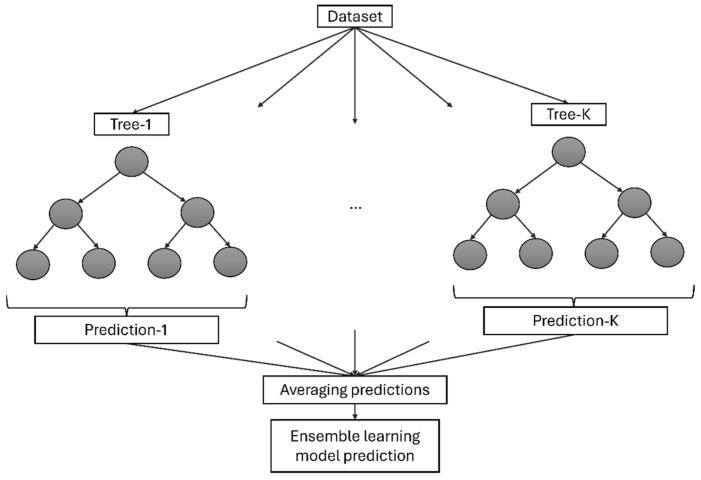
A general ensemble learning architecture based on decision trees.

Ensemble learning models are a powerful class of machine learning techniques that combine multiple individual models to produce a more accurate and robust prediction than any single model could achieve on its own [50]. By leveraging the diversity of the constituent models, ensemble methods can mitigate the weaknesses of individual models and exploit their complementary strengths. Bagging, boosting, and stacking are common techniques used in ensemble learning models to improve model performance [17]. In bagging, multiple models are trained independently on different subsets of the training data, and their predictions are combined through averaging or voting. Boosting sequentially trains a series of weak learners, with each subsequent model focusing on the examples that the previous models struggled with. Stacking involves training a meta-model that learns how to combine the predictions of multiple base models. Ensemble learning models are particularly effective when dealing with noisy or heterogeneous data [50]. In Figure 3, simplified general structure for ensemble learning models based on decision trees is illustrated.

#### 2.3.1. Random Forest

Random forest is an ensemble learning method based on bagging that constructs multiple decision trees during training and combines their predictions to make more accurate and robust predictions [51]. The key idea behind random forest is to introduce randomness both in the selection of the data samples used to train each tree and in the selection of features considered for splitting at each node. Each decision tree in the random forest is trained independently on a bootstrap sample of the training data, where samples are drawn with replacements [52]. Additionally, at each node of the tree, only a random subset of features is considered for splitting, ensuring that each tree in the forest learns different aspects of the data. The final prediction of the random forest is obtained by aggregating the predictions of all individual trees through averaging in regression or voting in classification tasks. Prediction *ŷ* of a new input *x* in a random forest model, for a regression task, is presented in Equation (19) [53].

(19) $$\hat{y}=\frac{1}{K}\sum_{i=1}^{K}T_{i}(x),$$

The *K* represents the number of trees in the forest and *T_i_*(*x*) represents the prediction of the *i*-th decision tree for input *x*. The random forest model combines multiple decision trees to produce accurate predictions while reducing overfitting. It effectively handles missing data and identifies influential features for interpretation [54]. Robust to outliers, it can handle large datasets efficiently but may struggle with imbalanced data and highly correlated features. However, random forest models are less interpretable and require tuning of multiple hyperparameters, leading to longer training times [51].

#### 2.3.2. Extreme Gradient Boosting (XGBoost)

Extreme gradient boosting (XGBoost) is an advanced implementation of gradient boosting, a powerful ensemble learning technique. Employing a supervised learning approach, it combines the predictions of multiple weaker models typically decision trees, in a sequential manner to accurately forecast an objective variable [15]. The key innovation of XGBoost lies in its optimization algorithm, which efficiently minimizes a regularized objective function by iteratively adding new trees to the ensemble [55]. XGBoost applies a gradient descent-based approach to minimize the loss function, incorporating both first-order gradients, i.e., gradients of the loss function with respect to predictions, and second-order gradients, i.e., gradients of the loss function with respect to the model parameters [15,56]. Thus, XGBoost can learn complex relationships in the data while preventing overfitting through regularization techniques such as shrinkage, which controls the contribution of each tree to the final ensemble, and tree pruning, which removes unnecessary branches from the model. The objective function *L* is minimized by XGBoost, as presented in Equation (20), where *l* is the loss function measuring the difference between the true label *y_i_*, the predicted label *ŷ*_i_, Ω(*f_k_*) is the regularization term penalizing the complexity of each tree *f_k_*, and *K* is the number of trees in the ensemble [56].

(20) $$L=\sum_{i=1}^{n}l(\hat{y_{i}},y_{i})+\sum_{k=1}^{K}\Omega (f_{k}),$$

XGBoost iteratively adds new trees to the ensemble to minimize this objective function, with each tree trained to correct the errors made by the previous ones [15]. XGBoost, known for its efficiency and scalability, often surpasses other machine learning algorithms in both speed and accuracy [55]. It incorporates built-in regularization techniques that add penalty terms to the loss function, such as L1 regularization (Lasso) and L2 regularization (Ridge) that add the absolute values of the coefficients and the squared values, respectively, to prevent overfitting and enhance generalization. Supporting various objective functions and evaluation metrics, XGBoost adapts well to regression, classification, and ranking tasks. It allows the derivation of feature importance scores to aid in feature selection and interpretation [57]. XGBoost handles missing values automatically and employs parallelization for faster training on large datasets. Advanced tree pruning techniques control model complexity, improving generalization performance. However, tuning numerous hyperparameters may require significant computational resources, and memory consumption can be high, limiting its applicability in memory-constrained environments [58]. Like other ensemble methods, XGBoost may sacrifice interpretability. Despite its regularization, overfitting may occur with complex datasets or inadequate tuning [15]. Sensitivity to outliers and challenges with scalability in very large datasets or distributed environments are potential limitations, and addressing imbalanced datasets may require additional techniques like class weighting or resampling [56].

### 2.4. Kernel Methods

Kernel methods are a class of machine learning techniques that encompass various approaches. Some kernel methods compute similarities between data points in the original input space without necessarily explicitly computing the transformation into a higher-dimensional feature space while some kernel models operate by implicitly mapping input data into a high-dimensional feature space using a kernel function [59]. This flexibility allows kernel methods to effectively capture complex patterns in data [60]. Kernel methods are especially useful when dealing with data that cannot be effectively linearly separated or modeled using traditional techniques [59].

#### 2.4.1. Support Vector Machine (SVM)

The SVM is a powerful supervised learning kernel model used for classification and regression tasks. In classification, SVMs aim to find the optimal hyperplane that separates data points belonging to different classes with the largest margin [61].

A SVM aims to find a function *f*(*x*) for a given set of training data points {*x_i_*, *y_i_*}, where *x_i_* is the input feature vector and *y_i_* is the corresponding continuous target value, such that the difference between the predicted and actual values is minimized, while still satisfying a specified margin *ϵ* [62]. Equation (21) presents objective function of SVM, where *w* is the weight vector orthogonal to the hyperplane, *b* is the bias term, *ξ_i_* and $\xi_{i}^{*}$ are slack variables representing the distance of the data point (*x_i_*, *y_i_*) from the margin, and *C* is the regularization parameter controlling the trade-off between maximizing the margin and minimizing the error [63].

(21) $$minimize\;\frac{1}{2}{\left\|w\right\|}^{2}+C\sum_{i=1}^{n}\left(\xi_{i}+\xi_{i}^{*}\right)\;\;\;subject\;to\;\left\{\begin{array}{c} \left|y_{i}-w\cdot x_{i}-b\right|\leq \epsilon +\xi_{i}\\ y_{i}-w\cdot x_{i}-b\leq \epsilon +\xi_{i}^{*}\\ -y_{i}+w\cdot x_{i}+b\leq \epsilon +\xi_{i}^{*} \end{array}\right.,$$

The kernel trick can also be applied to SVM to handle non-linear relationships between the input features and the target variable. SVMs excel in high-dimensional spaces, making them ideal for tasks like text classification and image recognition [63]. Their margin maximization objective mitigates overfitting, ensuring robust generalization to unseen data [61]. With different kernel functions, SVMs can handle both linearly and non-linearly separable data, offering flexibility in modeling complex relationships. By utilizing support vectors in the decision function, SVMs are memory-efficient and suitable for large datasets [63]. Even with small datasets, SVMs focus on support vectors near the decision boundary, enhancing generalization, and they also aim for global optimums, providing stable solutions compared to local optimization methods. Fine-tuning parameters like the regularization parameter *C* and kernel choice allows control over model complexity and performance [64]. However, parameter selection can be challenging, and training can be computationally intensive, especially with large datasets. Storing the entire training dataset in memory during training poses memory constraints, particularly with non-linear kernels or high-dimensional spaces, limits SVMs’ practicality for big data applications [62]. Additionally, SVMs lack interpretability due to their black-box nature and are sensitive to noisy data, potentially affecting performance, and extensions are needed for multi-class classification or regression tasks, adding complexity to the modeling process [61].

#### 2.4.2. Gaussian Processes (GPs)

GPs are probabilistic machine learning models that are categorized under kernel models in this paper due to their reliance on kernel functions to define the covariance structure between data points [65]. Unlike traditional kernel methods such as SVMs, which use kernel functions to implicitly map data points into a high-dimensional feature space, GPs directly model the distribution over functions using a covariance function [66]. This covariance function, also known as the kernel function, encodes assumptions about the relationships between input and output variables. Mean function *m*(*x*) and covariance function *k*(*x*, *x*′), where *x* and *x*′ are input points, fully specify a GP [67]. Given a set of observed data points {*x_i_*, *y_i_*}, where *y_i_* is the observed output corresponding to input *x_i_*, the joint distribution of the observed outputs *y* can be written as presented in Equation (22).

(22) y~N(m,K),

The elements of the covariance matrix *K* are computed using the covariance function *k*(*x_i_*, *x_j_*), which is often referred to as the kernel function [66]. Equation (23) illustrates the equation for computation of the output $y_{*}$ for a new input point $x_{*}$, where $\mu_{*}$ is the mean of the predictive distribution and $\sigma_{*}^{2}$ is its variance [66].

(23) $$P\left(\left.y_{*}\right|x_{*},x,y\right)=N(\mu_{*},\sigma_{*}^{2}),$$

The means of the predictive distribution μ_*_ and its variance σ_*_^2^ are computed as given in Equation (24) and Equation (25), respectively, where k_*_ is the vector of covariances between the new input x_*_ and the training inputs *x*, *K* is the covariance matric of the training inputs *x*, *σ_n_^2^* is the noise variance, *I* is the identity matrix, and the *k*(x_*_,x_*_) is the covariance between the new input x_*_ and itself [66].

(24) $$\mu_{*}=k_{*}^{T}{\left(K+\sigma_{n}^{2}I\right)}^{-1}y,$$

(25) $$\sigma_{*}^{2}=k\left(x_{*},x_{*}\right)-k_{*}^{T}{\left(K+\sigma_{n}^{2}I\right)}^{-1}k_{*}$$

GPs excel in modeling complex, non-linear relationships between variables across various data types, offering uncertainty estimates through confidence intervals, which is vital for decision-making under uncertainty [68]. GPs accommodate both interpolation and extrapolation, making them suitable for tasks with sparse or irregular data, and their adaptability to data complexity without manual hyperparameter tuning is a significant advantage of GP models [69]. As nonparametric models, GPs grow in accuracy with increasing data, facilitating robust regression and classification with Bayesian inference [67]. GPs can easily incorporate prior knowledge about the underlying process through the choice of covariance functions, enabling the integration of domain expertise into the modeling process; however, computational intensity poses challenges, especially with large datasets, as the need to store the entire dataset may be impractical [69]. GPs may struggle with high-dimensional or large-scale datasets due to computational constraints, impacting their applicability in signal analysis [67]. Additionally, kernel selection is crucial and may require domain expertise, while hyperparameter tuning influences model performance while also extending GPs to non-Gaussian likelihoods can be challenging [68]. Interpreting underlying processes may also be difficult due to their complexity and lack of explicit model parameters.

## 3. Preprocessing Requirements

Preprocessing is a critical step in preparing cerebral physiological signals for analysis using machine learning models, as it ensures the data are clean, structured, and well-suited to the chosen algorithm. Although the specific requirements vary, many preprocessing steps are shared across models, with slight adjustments based on the underlying assumptions and operations of each. For HMMs, preprocessing focuses on maintaining sequential data quality and interpretability. Noise reduction, such as bandpass filtering, is critical to isolating the frequencies relevant to the modeled states, while normalization ensures consistency across signal amplitudes [22,24]. Temporal segmentation into meaningful epochs (e.g., 1 s windows) is essential for capturing transitions between hidden states. Preprocessing may also include trend removal, such as detrending ICP signals, in order to focus on short-term dynamics and reduce the influence of long-term drift.

In CNNs, preprocessing often involves transforming time-series data into image-like representations, such as spectrograms or wavelet scalograms. Noise reduction and normalization are critical to ensure that visual features reflect meaningful physiological patterns [32,34]. Additionally, segmenting the data into fixed-size windows helps create consistent input sizes for the network. For temporal models like RNNs, LSTM networks, and ESNs, preprocessing emphasizes the preservation of temporal dependencies. Noise reduction and normalization are fundamental, while segmentation into sequences ensures that input data are structured consistently [37,42,47]. Sequence padding or truncation may also be required to handle varying sequence lengths effectively. Detrending is particularly relevant for signals like ICP or fNIRS, where non-stationary trends could interfere with learning temporal relationships.

Random forests and XGBoost, as tree-based models, are relatively robust to noise and outliers but still benefit from preprocessing to improve model interpretability and performance. Feature extraction and selection are often key steps for reducing dimensionality and focusing on relevant signal attributes, such as power in specific EEG frequency bands or oxygenation levels in fNIRS [70]. Normalization is less critical for these models but can improve feature importance metrics and consistency across datasets. For distance-based models like SVMs, proper scaling or normalization of features is essential, as these models rely on distance calculations for classification or regression [64]. Noise reduction and feature extraction are similarly crucial to reduce irrelevant variability and focus on patterns critical for decision boundaries. Outlier detection is especially important for SVMs, as extreme values can disproportionately affect the model’s performance.

Finally, GPs are highly sensitive to noise and outliers, making preprocessing crucial for reliable probabilistic modeling. Noise reduction, detrending, and outlier removal are fundamental to ensure that the GP models reflect true underlying processes rather than spurious variations [69]. Normalization helps standardize the data range, enabling smoother kernel function operations, while feature extraction can reduce dimensionality for computational efficiency.

Overall, preprocessing steps like noise reduction, normalization, segmentation, detrending, outlier handling, and feature extraction play a pivotal role in preparing data for analysis. Tailoring these steps to the specific model ensures that the algorithm can effectively capture and interpret the underlying dynamics of cerebral physiological signals.

## 4. Clinical Relevance

Studying and analyzing cerebral physiological signals is crucial for gaining a deeper understanding of brain function and dysfunction. The brain is the control center of the human body, responsible for regulating essential functions such as cognition, emotion, movement, and sensory perception. Cerebral physiological signals, such as EEG, and fNIRS provide valuable insights into the complex neural processes underlying these functions. By examining these signals, brain activity patterns, connectivity networks, and aberrant responses associated with various neurological conditions can be investigated. Understanding cerebral physiological signals is essential for diagnosing and monitoring disorders like epilepsy, Alzheimer’s disease, traumatic brain injury (TBI), and psychiatric illnesses. Additionally, it informs the development of novel therapies and interventions aimed at restoring or optimizing brain function, ultimately improving patient outcomes and quality of life.

Multivariate machine learning models play a crucial role in analyzing, modeling, and predicting cerebral physiologic signals in both human and veterinary cohorts. These models excel in handling the complexity and high dimensionality of cerebral data, integrating multiple variables simultaneously to uncover intricate patterns and relationships. In human studies, such models are utilized extensively in neuroimaging research, including EEG, and fNIRS data analysis, thus enabling the identification of biomarkers of neurological disorders, delineate brain networks, and predict disease progression or treatment outcomes [10,12,71,72,73,74,75,76,77,78,79,80,81,82,83,84,85,86,87,88,89,90,91,92,93,94,95,96,97,98,99,100,101]. For instance, in epilepsy research, multivariate machine learning models can distinguish between interictal and ictal states, aiding in seizure prediction and localization for potential surgical interventions [102,103,104,105]. The brain–computer interface (BCI) is the other important research area where multivariate machine learning models play a crucial role, since BCI analyzes signals from the brain, facilitating direct communication between humans and machines, and ultimately aiding individuals with severe disabilities in controlling external machines or robots to accomplish specific tasks [106,107,108,109]. Another research area involves the use of machine learning models to analyze high-frequency signals, e.g., EEG or electrocorticography (ECoG), in visual or auditory studies for tasks such as attention detection [110,111].

Similarly, in veterinary medicine, multivariate machine learning holds promise for understanding brain function and pathology in animals [112,113,114,115,116]. Multivariate machine learning models could allow analysis of complex cerebral data from veterinary cohort providing insights into disease mechanisms, optimizing treatment strategies, and improving prognostic accuracy [117].

Moreover, while these models support translational research, it is important to recognize that the outcomes observed in animal models cannot always be directly translated to human studies due to significant physiological and anatomical differences [118]. This highlights the need for careful consideration when applying findings from veterinary medicine to human brain disorders. Nevertheless, these models can still provide valuable insights and foster a collaborative approach to understanding brain disorders and developing innovative therapies for both humans and animals.

Multivariate machine learning models are instrumental in analyzing cerebral physiological signals, aiding in various research in both human and animal cohorts. Table 2 lists a compilation of studies employing non-linear multivariate machine learning models to analyze diverse cerebral physiological signals, spanning from EEG signals [39,104,108,119,120,121,122,123,124,125,126,127,128,129,130] to other cerebral physiologic signals like ICP and CPP [131,132,133,134], for tasks related to modeling and prediction. Overall, multivariate models have been used for tasks such as determining cerebral dynamic states; analyzing neuronal dynamics; detecting and classifying depression, fatigue, and emotions; investigating local field potential (LFP) states in Parkinson’s disease (PD); decoding movement intentions; classifying PD patients; classifying brain state changes; predicting brain age and hand kinematics; classifying preictal or interictal states; and carrying out feature extraction. Similarly, using other cerebral signals, tasks such as predicting ICP episodes, automatic sleep state scoring in neonates, distinguishing TBI patients, predicting neurological outcomes, and analyzing ICP and CPP signals have been carried out.

## 5. Limitations of the Models

Analyzing cerebral physiological data with multivariate machine learning models presents several challenges. Firstly, the high dimensionality of the data, often stemming from various imaging modalities or physiological sensors, complicates model construction and interpretation. Secondly, cerebral signals exhibit complex temporal dynamics and spatial interactions, necessitating sophisticated modeling techniques capable of capturing these intricate patterns effectively. Additionally, the presence of noise and artifacts in the data poses challenges for accurate inference, requiring robust preprocessing methods to enhance signal quality. Moreover, cerebral physiological data may suffer from missing values or irregular sampling rates, necessitating careful handling during data preprocessing and model training. Furthermore, the heterogeneity of cerebral signals across individuals, influenced by factors such as age, gender, and pathology, introduces variability that must be accounted for in model development and validation. Finally, the limited availability of labeled data and ground truth measurements complicates model validation and generalization to diverse populations or clinical settings.

Multivariate machine learning models, despite offering robust tools for analyzing complex data and making predictions, are encumbered by inherent limitations that affect their applicability and interpretability across diverse contexts. Estimating parameters in multivariate machine learning models can be difficult, particularly with high-dimensional data or complex relationships, potentially introducing biases in predictions. Furthermore, these models may be sensitive to initial conditions and have limited flexibility in capturing intricate variable interactions, thus limiting their usefulness. The computational complexity associated with training and analyzing multivariate machine learning models, coupled with the risk of overfitting and interpretability challenges, poses significant obstacles to their application.

However, it is important to acknowledge that specific models may have unique limitations and drawbacks. For instance, HMMs may struggle to capture the complex temporal dynamics inherent in cerebral signals due to their assumption of discrete latent states. CNNs, designed for spatial hierarchies, may overlook the temporal dependencies crucial in cerebral signal analysis. RNNs and LSTMs are adept at modeling sequential data, yet they might encounter difficulties in handling long-term dependencies or noisy signals. GPs, although effective in uncertainty quantification, may face scalability issues with large datasets. SVMs, random forests, and XGBoost, while robust and versatile, may lack interpretability in complex cerebral signal patterns. Furthermore, selecting the appropriate model architecture, tuning hyperparameters, and ensuring generalization to unseen data still pose significant challenges in cerebral physiologic signal analysis. Therefore, careful consideration of the strengths and limitations of each model is essential for effective utilization in this domain. Addressing the challenges stemming from cerebral physiological data with respect to limitations of multivariate machine learning models requires a multidisciplinary approach, integrating expertise from neuroscience, signal processing, and machine learning to develop robust and interpretable models for cerebral physiological data analysis.

## 6. Conclusions

Studying cerebral physiological signals is essential for understanding brain function and dysfunction. Biomedical sensors like EEG and fNIRS provide insights into neural processes, aiding in the diagnosis and treatment of neurological disorders such as epilepsy, Alzheimer’s disease, and TBI. Multivariate machine learning models are crucial in this field, capable of handling complex and high-dimensional data recorded by these sensors to uncover patterns and predict outcomes. These models are used extensively in neuroimaging research to identify biomarkers, delineate brain networks, and forecast disease progression or treatment efficacy. Additionally, they play a pivotal role in BCIs, which, through the use of real-time sensor data, enable direct communication between the brain and external devices, helping individuals with severe disabilities.

However, analyzing cerebral physiological data with these models presents challenges, such as high dimensionality, noise, artifacts, and variability across individuals. Different models have unique limitations and strengths that must be carefully considered for the successful application of these models in cerebral physiological signal modeling and prediction. Importantly, the use of machine learning models in this domain is still in its infancy, and achieving their full potential requires a concerted effort to address foundational issues. Standard sensor technologies and imaging modalities must be utilized to establish reliable datasets and ensure consistency in data collection and preprocessing. These steps are critical for accurately categorizing disease states and establishing a robust foundation for subsequent predictive modeling. Equally important is the selection of the most appropriate machine learning algorithms for specific tasks, taking into account their ability to manage the unique challenges of cerebral data, such as its multivariate nature and person-specific differences. Accreditation and regulatory frameworks should be developed and adhered to, ensuring that these models meet clinical and ethical standards.

Ultimately, the integration of standardized practices, ideal algorithms, and accredited frameworks will enable machine learning models to deliver on their promise of improving prognostic accuracy and enhancing treatment strategies. By addressing these challenges, machine learning has the potential to revolutionize our understanding of brain function and dysfunction, enabling the development of innovative diagnostic tools and personalized therapies for neurological disorders.

## Figures and Tables

**Table 1:** Summary of the non-linear state-space models.

Model	Description	Advantages	Disadvantages
HMM	Probabilistic graphical model used to model sequential data, such as recorded data, by considering both directly measured factors (observable) and underlying aspects that cannot be directly seen (hidden), such as disease states.	Flexibility for modeling diverse sequential data types. Ability to capture temporal dependencies and transitions. Interpretability, providing insights into hidden state dynamics. Feature extraction capabilities for capturing relevant patterns. Well-suited for analyzing sequential data in various domains.	Assumption of Markovian property may not hold for complex systems. Fixed state space can be challenging when the number of states is unknown. Limited modeling of long-term dependencies in data. Difficulty with high-dimensional data and computational complexity. Sensitivity to initialization and parameter tuning. Inference complexity increases with large state spaces or long sequences. Limited representation power compared to deep learning models. Difficulty in handling continuous data without preprocessing. Vulnerability to overfitting, particularly with large state spaces relative to data size.
CNN	Neural network architecture effective at capturing spatial hierarchies of features within data.	Hierarchical feature learning captures progressively complex features. Translation invariance enables robustness to spatial variations. Sparse connectivity reduces parameters and computational load. Parameter sharing facilitates generalization and handling of variable inputs. Effective for high-dimensional data like images and videos. Parallelizable operations enable fast training and inference. Transfer learning accelerates training with pre-trained models. Interpretability through visualization aids in model understanding. Improved fundamental feature extraction.	Limited interpretability of learned features. Requirement for large amounts of labeled data. Sensitivity to variations in hyperparameters. Lack of spatial context understanding in some cases. Difficulty in handling irregular data structures. Complexity of model architecture design. Vulnerability to adversarial attacks, meaning subtle alterations to input data can lead the network to confidently misclassify. Heavy computational requirements for training and inference.
RNN	Neural network architecture designed for processing sequential data by allowing connections between units to form directed cycles, enabling information persistence over time.	Temporal dynamics for time-series prediction and sequence tasks. Ability to process variable-length inputs. Shared parameters facilitate learning of long-term dependencies. Natural representation for sequential data tasks. Stateful memory captures context across time steps. Gradient propagation commonly with backpropagation through time.	Vanishing and exploding gradients hinder training. Short-term memory limits capture of long-range dependencies. Difficulty in capturing long-term dependencies. Sequential computation slows training and inference. Sensitivity to hyperparameters affects performance. Training instability with large datasets or complex architectures. Inadequacy in capturing complex contextual information.
LSTM	Type of RNN architecture designed to address the vanishing gradient problem and capture long-term dependencies by introducing specialized memory cells with gating mechanisms.	Capable of capturing long-term dependencies in sequences. Addresses the vanishing gradient problem for stable training. Utilizes gating mechanisms for better control over information flow. Maintains stateful memory for retaining relevant context. Versatile and effective for various sequential data tasks including time-series prediction.	Increased complexity and computational requirements. Proneness to overfitting, especially with limited data. Sensitivity to hyperparameters, requiring careful tuning. Difficulty in interpreting internal mechanisms. Limited memory for processing very long sequences. Potential gradient explosion during training.
ESN	Type of RNN model that utilizes a fixed, randomly connected reservoir and only trains the output weights, making them efficient for processing temporal data.	Training only the output layer is fast and computationally inexpensive. The fixed reservoir simplifies the network design and reduces the parameters to optimize. The reservoir transforms inputs into a high-dimensional space with diverse dynamic behavior. Fewer trainable parameters lower the risk of overfitting. Fixed reservoir weights provide stable and predictable dynamics. Applicable to various tasks involving temporal data, such as time series prediction and signal processing. Not affected by vanishing or exploding gradient issues.	Sensitivity to reservoir size, connectivity, and spectral radius. Untrained reservoir may not suit specific input data characteristics. Large reservoirs consume significant memory resources. Fixed reservoir cannot adapt to new data patterns during training. Challenging to find the optimal reservoir configuration for specific tasks.
Random forest	An ensemble learning method that constructs multiple decision trees during training and outputs the mode of the classes (classification) or the mean prediction (regression) of the individual trees.	High accuracy through the aggregation of multiple decision trees. Robustness to overfitting compared to individual trees. Effective handling of missing data. Provision of feature importance measures for interpretation. Ability to capture non-linear relationships. Robustness to outliers in the data. Efficiency in handling large datasets and high dimensionality.	Less interpretability compared to simpler models. Computational complexity can lead to longer training times. Memory-intensive due to storing multiple trees. Bias towards majority classes in imbalanced datasets. Time-consuming hyperparameter tuning. Slower prediction speed compared to simpler models for real-time applications. Reduced effectiveness with highly correlated features.
XGBoost	An implementation of random forest algorithms designed for efficiency, speed, and accuracy in supervised learning tasks.	High performance with efficiency and scalability. Built-in regularization techniques for preventing overfitting. Flexibility in supporting various objective functions and metrics. Feature importance scores for informed feature selection. Automatic handling of missing values in the data. Parallelization for faster training on large datasets. Advanced tree pruning for improved model complexity control. Useful in feature extraction.	Complexity in hyperparameter tuning. High memory usage with large datasets or deep trees. Reduced interpretability as a black box model. Potential overfitting, especially with complex datasets. Sensitivity to outliers in the data. Scalability limitations with extremely large datasets. Challenges in handling imbalanced datasets.
SVM	A supervised machine learning algorithm used for classification and regression tasks, aiming to find the optimal hyperplane that best separates different classes or predicts continuous values.	Effective in high-dimensional spaces. Robust to overfitting due to margin maximization. Versatility with linear and non-linear kernel functions. Memory efficiency using a subset of support vectors. Effective with small datasets, focusing on support vectors. Aim to find global optimum for more stable solutions. Controlled complexity through parameter tuning.	Sensitivity to parameter tuning. Computationally intensive for large datasets. Memory-intensive storage of entire training dataset. Difficulty with scalability to very large datasets. Limited interpretability as black-box models. Performance degradation with noisy data. Inherent limitation to binary classification tasks.
GP	A probabilistic model that defines a distribution over functions, where any finite set of points has a joint Gaussian distribution.	Flexibility to model complex relationships without assuming specific functional forms. Uncertainty quantification for reliable predictions under uncertainty. Capable of interpolation and extrapolation for sparse or irregularly sampled data. Automatic adjustment of complexity based on available data. Nonparametric nature allows for growing complexity with increasing data. Probabilistic framework enables principled uncertainty estimation and Bayesian inference. Easy incorporation of prior knowledge through choice of covariance functions.	Computational complexity for large datasets. Memory requirements for storing entire datasets. Limited scalability to high-dimensional data. Challenging choice of covariance function. Sensitivity to hyperparameters. Difficulty with non-Gaussian likelihoods. Limited interpretability due to complex nature.

CNN, convolutional neural network; ESN, echo state networks; GP, Gaussian process; HMM, hidden Markov model; LSTM, long short-term memory; RNN, recurrent neural network; SVM, support vector machines; XGBoost, extreme gradient boosting.

**Table 2:** Studies employing non-linear multivariate state-space models for various cerebral physiological signals.

Study	Study Group	Relevant ML Model	Cerebral Physiology	Significance of the Model in the Study
Asgari et al., 2019 [131]	Adult patients with TBI	HMM	ICP, CPP, PRx, RAP Other: ABP	A HMM was utilized to determine the cerebral dynamic states with respect to various cerebral physiological signals.
Farhadi et al., 2019 [132]	Pediatric ICU patients	SVM, random forest	ICP, CPP Other: MAP, HR, BP	SVM and random forest models were compared with other models for prediction of ICP episodes, with the random forest model achieving the highest prediction accuracy.
Fraiwan and Alkhodari, 2020 [119]	Neonates	LSTM	Sleep-state EEG recordings	A LSTM algorithm was utilized and compared with studies from the literature for automatic sleep state scoring for neonates, achieving the highest accuracy.
Güiza et al., 2013 [133]	Patients with TBI	GP	ICP, CPP Other: MAP	A GP was compared to logistic regression for increased ICP episode prediction and early prediction of unfavorable neurological outcome, with the GP model exhibiting the best overall performance.
Itälinna et al., 2023 [125]	Patients with mild TBI and healthy controls	SVM	MEG	A SVM classifier was trained on quantitative deviation maps to distinguish TBI patients from healthy control subjects.
Jiang et al., 2023 [126]	Healthy volunteers and individuals with major depressive disorder	HMM with multivariate autoregressive observation (MAR)	Resting-state and task-state EEG recordings	The HMM-MAR model illustrated the ability to capture neuronal dynamics from EEG signals and to interpret brain disease pathogenesis by analyzing state transitions.
Khadidos et al., 2023 [120]	Healthy volunteers and patients with depression	Decision tree, random forest, CNN, RNN, LSTM, XGBoost	Stimuli-induced EEG recordings	Models were compared for detection and classification of depression. CNN showed the best performance among all employed models.
Khawaldeh et al., 2022 [97]	PD patients	HMM	LFP	A HMM was used to detect different LFP states to investigate the impact of various spectral states in the subthalamic nucleus LFP on motor impairment in PD patients.
Kim and Jeong, 2019 [124]	Healthy volunteers	ESN and Gaussian readouts	Stimuli-induced EEG recordings	ESN and Gaussian readouts were shown to effectively decode user movement intentions using a low-cost, portable EEG system.
Lee et al., 2021 [135]	Healthy volunteers and PD patients	CNN-RNN	Resting-state EEG recordings	CNNs were employed for feature extraction, while RNN model was used for detection and classification of PD patients, showing better performance compared to baseline machine learning models as well as the deep learning models from the literature.
Mughal et al., 2022 [39]	Healthy volunteers	CNN-LSTM	fNIRS and task-state EEG recordings	The CNN-LSTM hybrid model was applied to images generated by recurrence plots for stand-alone EEG and fNIRS data, as well as hybrid EEG-fNIRS data, for the classification of changes in brain state. The performance of the model using hybrid EEG-fNIRS data were observed to be superior compared to the other two image sets, as well as to the results reported in the literature.
Myers et al., 2016 [134]	Patients with severe TBI	GP	ICP, PbtO_2_ Other: MAP, EtCO_2_, SaO_2_	A GP was compared to logistic regression and autoregressive model for univariate and multivariate prediction of ICP and PbtO_2_.
Nadalizadeh et al., 2024 [127]	Drivers in fatigued and normal states	k-NN, SVM, random forest	Resting-state and task-state EEG recordings	k-NN, SVM, and random forest classifiers were applied to features extracted from EEG signals for fatigue detection and recognition.
Paliwal et al., 2024 [128]	Patients of various ages who had undergone routine clinical EEG scans	CNN	EEG	A CNN was used to predict brain age of a patient from EEG scans. CNN model performance was shown to improve with multivariate iterative filtering.
Pancholi et al., 2023 [108]	Healthy volunteers	MLP, CNN-LSTM, WPD CNN-LSTM	Task-state EEG recordings	MLP, CNN-LSTM, and WPD CNN-LSTM are employed and compared for the prediction of hand kinematic trajectory.
Uyulan et al., 2022 [129]	Patients with major depressive disorder and healthy volunteers	Pretrained CNN-LSTM	Resting-state EEG recordings	A hybrid model was employed alongside a stand-alone LSTM to detect depression-specific information from EEG signals for depression classification. The hybrid model demonstrates better performance, lower training time, and no overfitting issues.
Williamson et al., 2012 [104]	Patients with medically intractable focal epilepsy	SVM	Intracranial EEG	A SVM model was trained on 15 s of EEG signals for the classification of preictal or interictal state of patients.
Yang et al., 2024 [123]	Healthy volunteers	M-ESN, ESN	Stimuli-induced EEG recordings	A M-ESN, where ESN hidden state is directly initialized, outperformed standard ESN while having a smaller reservoir size and a simpler training process.
Xing et al., 2022 [121]	Healthy volunteers	CNN-LSTM	Stimuli-induced EEG recordings	A CNN-LSTM model was utilized for emotion detection by combining spatial–frequency–temporal features extracted from EEG signals. The model showed better performance compared to baseline methods.
Zafar et al., 2017 [130]	Healthy volunteers	CNN	Stimuli-induced EEG recordings	A CNN was utilized for feature extraction from EEG signals, which were then used in a separate classification task.
Zong et al., 2023 [122]	Healthy volunteers	XGBoost	Stimuli-induced EEG recordings	XGBoost was employed for emotion recognition task using features extracted with a feature attention network module. The proposed model was shown to have better performance than baseline models.

ABP, arterial blood pressure; BP, blood pressure; CNN, convolutional neural network; CPP, cerebral perfusion pressure; EEG, electroencephalography; ESN, echo state network; EtCO_2_, end-tidal carbon dioxide; fNIRS, functional near-infrared spectroscopy; GP, Gaussian process; HMM, hidden Markov model; HR, heart rate; ICP, intracranial pressure; ICU, intensive care unit; LFP, local field potential; LSTM, long short-term memory; M-ESN, modular echo state network; MAP, mean arterial blood pressure; MAR, multivariate autoregressive; MEG, magnetoencephalography; ML, machine learning; MLP, multi-layer perceptron; PbtO_2_, cerebral tissue oxygenation; PD, Parkinson’s disease; PRx, pressure reactivity index; RAP, pressure–volume reserve; RNN, recurrent neural network; SaO_2_, arterial oxygen saturation; SVM, support vector machines; TBI, traumatic brain injury; WPD, wavelet packet decomposition; XGBoost, extreme gradient boosting.

## Data Availability

No new data were created or analyzed in this study. Data sharing is not applicable to this article.
